# Double inlay plus ventral onlay buccal mucosa graft for simultaneous penile and bulbar urethral stricture

**DOI:** 10.1590/S1677-5538.IBJU.2017.0067

**Published:** 2018

**Authors:** Luciano A. Favorito, Paulo P. Conte, Ulisses G. Sobrinho, Rodrigo G. Martins, Tomas Accioly

**Affiliations:** 1 Seção de Urologia, Hospital Federal da Lagoa - Rio de Janeiro, RJ, Brasil

## Abstract

**Objectives:**

Buccal mucosa grafts and fascio-cutaneous flaps are frequently used in long anterior urethral strictures (1). The inlay and onlay buccal mucosa grafts are easier to perform, do not need urethral mobilization and generally have good long-term results (2–4). In the present video, we present a case where we used a double buccal mucosa graft technique in a simultaneous penile and bulbar urethral stricture.

**Materials and Methods:**

A 54 year-old male patient was submitted to appendectomy where a urethral catheter was used for two days in May 2015. Three months after surgery, the patient complained of acute urinary retention and a supra-pubic tube was indicated. Urethrocystography was performed two weeks later and showed strictures in penile and bulbar urethra with 3.5 cm and 3 cm in length respectively. Urethroplasty was proposed for the surgical treatment in this case. We used a perineal approach with a ventral sagittal urethrotomy in both strictures. Penile urethra stricture measuring 3.5 cm in length was observed and a free graft from the buccal mucosa was harvested and placed into the longitudinal incision in the dorsal urethra and fixed with interrupted suture as dorsal inlay. Bulbar urethra stricture measuring 3 cm was observed and a free graft from the buccal mucosa was harvested and placed into the longitudinal incision in the ventral urethra and fixed with interrupted suture as ventral onlay. The ventral urethrotomy was closed over a 16Fr Foley catheter and the skin incision was then closed in layers.

**Results:**

No intraoperative or postoperative complications occurred. The patient could achieve satisfactory voiding and no complication was seen during the six-month follow-up. Postoperative imaging demonstrated a widely patent urethra, and the mean peak flow was 12 mL/s.

**Conclusion:**

The BMG placement can be ventral, dorsal, lateral or combined dorsal and ventral BMG in the meeting of stricture but the first two are most common (5, 6). Ventral location provides the advantages of ease of exposure and good vascular supply by avoiding circumferential rotation of the urethra (7). Early success rates of dorsal and ventral onlay with BMG were 96 and 85%, respectively. However, long-term follow-up revealed essentially no difference in success rates (8–11). Anterior urethral stricture treatments are various, and comprehensive consideration should be given in selecting individualized treatment programs, which must be combined with the patient's stricture, length, complexity, and other factors. Traditionally, anastomotic procedures with transection and urethral excision are suggested for short bulbar strictures, while longer strictures are treated by patch graft urethroplasty preferably using the buccal mucosa as gold-standard material due to its histological characteristics. The current management for complex urethral strictures commonly uses open reconstruction with buccal mucosa urethroplasty. However, there are multiple situations whereby buccal mucosa is inadequate (pan-urethral stricture or prior buccal harvest) or inappropriate for utilization (heavy tobacco use or oral radiation). Multiple options exist for use as alternatives or adjuncts to buccal mucosa in complex urethral strictures (injectable antifibrotic agents, augmentation urethroplasty with skin flaps, lingual mucosa, colonic mucosa, and new developments in tissue engineering for urethral graft material). In the present case, our patient had two strictures and we chose to correct the first stricture with a dorsal graft and the bulbar stricture with a ventral graft because of our personal expertise. We can conclude that the double buccal mucosa graft is easier to perform and can be an option to repair multiple urethral strictures.

## ARTICLE INFO

**Available at: http://www.intbrazjurol.com.br/video-section/20170067_Favorito_et_al**

**Int Braz J Urol. 2018; 44 (Video #9): 838-9**
